# Colour pattern predicts outcome of female contest competition in a sexually monomorphic fish

**DOI:** 10.1098/rsbl.2018.0480

**Published:** 2018-11-07

**Authors:** Angelika Ziegelbecker, Florian Richter, Kristina M. Sefc

**Affiliations:** Institute of Biology, University of Graz, Universitätsplatz 2, 8010 Graz, Austria

**Keywords:** female competition, social selection, colour pattern, sexual monomorphism, Cichlidae, *Tropheus*

## Abstract

Selection arising from social competition over non-mating resources, i.e. resources that do not directly and immediately affect mating success, offers a powerful alternative to sexual selection to explain the evolution of conspicuous ornaments, particularly in females. Here, we address the hypothesis that competition associated with the territoriality exhibited by both males and females in the cichlid fish *Tropheus* selects for the display of a conspicuous colour pattern in both sexes. The investigated pattern consists of a vertical carotenoid-coloured bar on a black body. Bar width affected the probability of winning in size-matched female–female, but not male–male, contests for territory possession. Our results support the idea that the emergence of female territoriality contributed to the evolution of sexual monomorphism from a dimorphic ancestor, in that females acquired the same conspicuous coloration as males to communicate in contest competition.

## 1. Introduction

The evolution of sexually monomorphic ornaments and armaments is often explained by mutual mate choice or competition for mating opportunities in both sexes [1]. Alternatively, it has been argued that in comparison to sexual selection, competition over non-sexual resources (i.e. other than mates) is more likely to affect both sexes similarly and hence underlie monomorphism in competitive traits [2,3]. While sexually monomorphic traits do not necessarily serve the same functions in males and females [4], several studies have indeed demonstrated correlations between body coloration and dominance in both sexes [5–9]. Yet, competition in non-sexual situations, such as during dominance interactions, can still directly influence mating success [7,8,10,11]. One solution to reduce the ambiguity over the types of benefits gained from competitive success is to study competition outside the breeding season [12]. Or, if no discrete breeding seasons exist for a given taxon, as in the current study, another solution is to examine female competition over resources that do not confer reproductive benefits immediately or over the short-term.

In the cichlid fish genus *Tropheus*, endemic to Lake Tanganyika, both males and females compete for individual feeding territories and use body colour signals to communicate social status and motivation in competitive and courtship interactions [13]. Spawning takes place in the males' territories; a female will join the male on his territory for several days to weeks, over which time she feeds intensely and then spawns. The female then leaves the male's territory to provide sole maternal mouthbrooding, after which she establishes her own feeding territory, where she remains for the duration of her interbrood interval (several months) [13]. Females compete (with males and females) to establish their own feeding territories, whereas the male-biased sex ratio [14] keeps female competition over mates low. While the quality, i.e. the structure, of a male's territory influences female mate choice [15], the quality of a female's feeding territory does not *immediately* influence her mating success.

**Figure 1.**
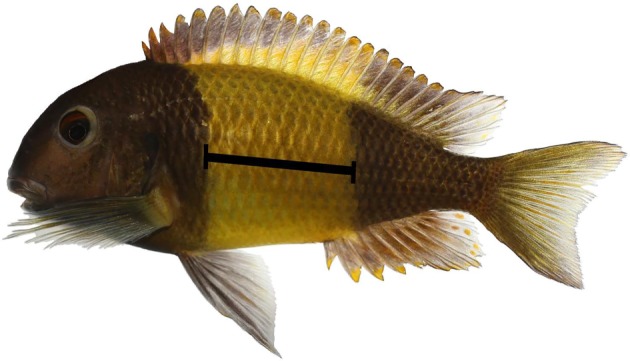
*Tropheus* sp. ‘black’, population Ikola. Bar width was measured along the lower lateral line (black bar). (Online version in colour.)

Cichlid lineages basal to *Tropheus* [16] are sexually dimorphic, with inconspicuous, small and non-territorial females. We hypothesize that the evolution of the male-like phenotypes in female *Tropheus* is linked to competition for feeding territories. In particular, the trophic specialization on epilithic algae [13] could have promoted territoriality in both sexes [17] and exposed females to selection on traits associated with resource holding potential such as body size [18] or coloration. Here, we test the prediction that the geographically variable, but sexually monomorphic colour patterns of *Tropheus* influence both female–female and male–male contest competition. The tested colour pattern is the width of the carotenoid-coloured yellow bar on a black body (figure 1), displayed by *Tropheus* sp. ‘black’ from Ikola, Tanzania. We predicted that bar width could be either negatively or positively correlated with dominance, depending on whether dominance is related to the black, melanin-coloration of the body, or to the yellow, carotenoid-coloration of the bar [19].

## 2. Material and methods

Territorial contests, in which two fish competed for a territory furnished with a brick structure (electronic supplementary material, figure S1), were staged between approximately size-matched, same-sex opponents (17 male–male and 18 female–female contests; each fish used only once) and videotaped. Winners were identified by continuous occupation of the bricks and the display of dominant coloration (intense black and yellow; electronic supplementary material, figure S2). We scored contest duration (first interaction until establishment of unchallenged dominance) and identity of the winner. Using photographs, the width of the yellow bar (figure 1) was quantified in relation to standard length (SL), both measured to the nearest 1 mm. Relative differences in body size (RSD) between contestants were expressed as (SL_focal fish_ – SL_opponent fish_)/(SL_focal fish_ + SL_opponent fish_). Relative differences in bar width (RBD) were calculated similarly. Body condition factor (CF) was measured as the residuals from a log(weight) against log(SL) regression and condition factor differences (CFD) between contestants were calculated as CFD = CF_focal fish_ − CF_opponent fish_. Body size and bar width were measured from all available fish (*n* = 77), 70 of which were used in the contest experiment. Additionally, we measured 44 of these fish multiple times over a period of up to approximately 600 days to monitor changes in bar width over time.

Detailed descriptions of experimental procedures and statistical analyses are provided in the electronic supplementary material. Generalized and general linear models were used to test for effects of RBD, RSD and CFD on contest outcome and duration. Analyses were run in R v. 3.1.2.

## 3. Results

Bar width (scaled by dividing by SL) was not correlated with SL (Pearson's *r* = −0.05, *p* = 0.65, *N* = 77) and slightly bigger in females (36.0% of SL) than in males (34.6% of SL; *t* = 1.9, *p* = 0.05, *N* = 77). Intra-individual variation in bar width over periods of up to approximately 600 days was small compared to among-individual variation (proportion of variance among individuals: *ω*^2^ = 0.91; *F* = 23.77, *p* < 0.001, *N* = 44 fish; electronic supplementary material, figure S3).

**Figure 2.**
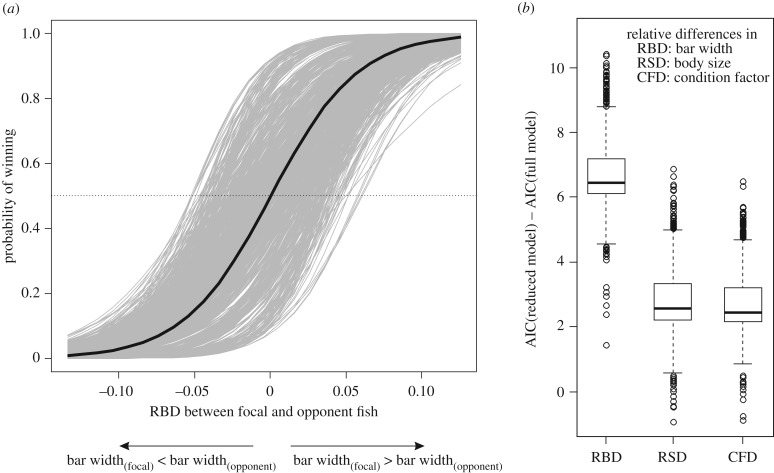
Effect of bar width differences on the probability of winning in female–female contests. (*a*) The arbitrary designations of contestants as ‘focal’ and ‘opponent’ were randomized to produce 731 permuted datasets. Logistic regression models estimated the effect of RBD on the probability of winning, while accounting for RSD and CFD, for each permuted dataset (grey lines). Black line: mean across the permutated datasets; dotted line: equal probability of winning and losing. (*b*) Comparison of model AIC values. One factor at a time was dropped from the full model (contest outcome ∼ RBD + RSD + CFD), and boxplots show the variation of ΔAIC in the permuted datasets.

In female–female contests, but not in male–male contests, winners had wider bars than their opponents on average (table 1). Wider bars (i.e. larger RBD) increased the likelihood of winning in female–female contests when controlling for RSD and CFD (figure 2). Body size and condition did not differ significantly between winners and losers in both sexes (table 1).

**Table 1.** Differences in bar width (RBD), body size (RSD) and condition (CFD) between winners and losers in female and male contests. *β*_0_: intercepts in general linear models with one of the three factors (RBD, RSD or CFD; all mean-centred and scaled) as dependent variable, sex of contestants as predictor and the other two factors as covariates in interaction with sex. *, *p* < 0.05.

dependent variable	female contests	male contests	sex difference
RBD	*β*_0_ = 0.039, *p* = 0.014*	*β*_0_ = −0.010, *p* = 0.517	*p* = 0.029*
RSD	*β*_0_ = 0.004, *p* = 0.182	*β*_0_ = 0.005, *p* = 0.126	*p* = 0.871
CFD	*β*_0_ = 0.002, *p* = 0.906	*β*_0_ = 0.007, *p* = 0.683	*p* = 0.835

Contest duration (median: 50 s, mean: 106 s, maximum: 927 s) did not differ significantly between the sexes, but was negatively correlated with asymmetries in bar width (i.e. absolute values of RBD) in female–female contests (table 2).

**Table 2.** Contest duration in male and female contests. Absolute values of RBD, RSD and CFD represent the extent of asymmetry between contestants in a trial. Non-significant interactions were dropped from the general linear model. Contest duration was square-root-transformed. **, *p* < 0.01; *, *p* < 0.05.

model: √duration ∼ |RBD| : sex + |RSD| + |CFD|	estimate (*β*)	s.e.	*p*-value
|RBD| : sex	64.0	27.65	0.030*
|RBD| in female–female contests	−51.0	17.97	0.009**
|RBD| in male–male contests	13.0	19.72	0.515
|RSD|	−24.8	51.32	0.633
|CFD|	3.0	11.73	0.798

## 4. Discussion

The contest experiment revealed a competitive advantage for females with wide yellow bars, in terms of both contest outcome and duration, which is consistent with the hypothesis that females acquired their conspicuous coloration for communication in competitive contexts. Given that melanin, i.e. dark, patch size is associated with dominance in some taxa [19,20], a reverse effect of bar width might actually have been expected, as more black is displayed by fish with narrower yellow bars. In several bird species, dominance is predicted by the size of carotenoid-coloured plumage patches and bare parts [5,6,9,21]. Whereas most plumage traits reflect past condition during feather growth, the size of avian bare parts such as shields can dynamically respond to changes in body condition and social environment [21]. In the adult *Tropheus* ‘Ikola’, the width of the yellow bar, which is associated with variation in melanophore density (electronic supplementary material, figure S4), remained constant over long time intervals and may be determined during maturation and formation of the adult colour pattern [22]. Rather than exposing current condition, both adult colour pattern and physiological performance may be influenced by early-life conditions, as has already been demonstrated in other animals [23,24]. Any link between colour pattern and physiological condition allows contestants to assess each other's fighting ability in order to avoid or curtail dangerous fights [19]. The observed correlations between RBD and both contest outcome and duration, in female–female contests, suggest covariation between bar width and fighting ability. But whether bar width functions as a status signal remains unclear based on current data. Importantly, while bar width is a fixed trait in *Tropheus* ‘Ikola’, physiological colour changes allow these fish to adjust their colour *contrasts* quickly, i.e. within seconds, to variation in the social environment. For instance, the yellow bar appears less pronounced and less expansive when a fish is subordinate as opposed to when it is dominant (electronic supplementary material, figure S2). Given communication via physiological modifications of the colour pattern, a signalling function of the morphological variation in bar width is not unlikely.

The phylogenetic background of *Tropheus* implies an ancestral condition of sexual dimorphism with colourful, territorial males and drab-coloured, non-territorial females [16]. In a previous experiment, body size affected contest outcome equally in both sexes, supporting a role of territorial competition in the evolution of sexual size monomorphism [18]. Although the present study detected no connection between bar width and contest outcome in males, the conspicuous colour pattern might still mediate male competition through variation in intensity and contrast. By identifying a competitive function of the female colour pattern, our study supports the hypothesis that following the transition to female territoriality, competition over a non-mating resource entailed a need for colour-based communication and promoted the expression of male-like colour patterns in female *Tropheus*. Our empirical data contribute to the longstanding interest in the evolution of female ornamentation and sexual monomorphism in visual showiness [2,3,11,25].

## Supplementary Material

Methods and supplementary figures

## Supplementary Material

Supplementary data 1

## Supplementary Material

Supplementary data 2

## Supplementary Material

Supplementary data 3

## Supplementary Material

Supplementary data 4

## Data accessibility

The datasets supporting this article have been uploaded as part of the supplementary material.
