# Supplementary material for: Colour pattern predicts outcome of female contest competition in a sexually monomorphic fish
Source: Biol Lett. 2018 Nov 7;14(11):20180480. doi: 10.1098/rsbl.2018.0480 (PMC6283925; doi:10.1098/rsbl.2018.0480)

**Supplementary Materials**

**Colour pattern predicts outcome of female contest competition in a sexually monomorphic fish**

Angelika Ziegelbecker, Florian Richter, Kristina M. Sefc

**Biology Letters**

**Supplementary Material and Methods**

**Fish husbandry**

Wild-caught adult *Tropheus sp. ‘black’* of the population ‘Ikola’ (Tanzania) were imported by an ornamental fish trader and kept in mixed stock tanks. Two weeks before the experiment, fish were separated by sex and housed individually (tank dimensions, LxWxD 60x35x30 cm). All tanks were filtered with internal box filters and illuminated with overhead white light on a 12:12 h light/dark cycle. Temperature was kept between 24 and 27°C. Size matched fish selected as opponents in the subsequent contest trials were fed equal amounts of food (100-300 mg algae flake food per day, depending on their size). After completion of the experiment, fish were returned to male and female stock tanks.

**Body size, condition factor and bar width measurements**

Standard length (SL) of fish was measured to the nearest 1 mm. On the day before being used in a contest trial, fish were weighed to the nearest 0.1 g. The condition factor was measured as residuals of log(weight) on log(SL) (Fechhelm et al. 1995).

The width and area of the yellow bar was measured from photographs in ImageJ vs. 1.x. For photography (camera model: Canon Powershot G11), fish were gently held against the glass front of a dedicated photography tank. Initially, we took four colour bar measures: upper bar width (along the row of scales perforated by the upper lateral line), middle bar width (along the row of scales perforated by the lower lateral line), lower bar width (along the ventral edge of the body), and area. The measurements of middle bar width were found to have the best reproducibility of the three bar width measures and a high correlation with bar area (r=0.86, p << 0.001, N=70 fish). Middle bar width measured on the left side of the fish was therefore used to quantify the color pattern in the subsequent analyses.

Body size and bar width measurements were taken from a total of 77 fish. From 44 of these fish, we took several measurements over a period of up to 600 days and monitored the change in relative bar width (bar width divided by SL) over time. 70 of the 77 fish (34 males and 36 females) were used in the contest competition experiment.

**Contest competition experiment**

Four contest arenas were installed by dividing each of 2 large tanks (LxWxD 150x70x50 cm) into two experimental compartments (dimensions Length x Width x Water Depth = 46x50x42 cm) using opaque plastic boards. Within tanks, arenas were separated by a central compartment containing internal box filter and heater.

Before each contest trial, the arena was partitioned in two halves by a wire mesh (mesh size 13 mm) and an opaque plastic board. Four clay bricks were placed in the arena to form a cave that was bisected by these dividers (figure S1). This arrangement allowed each of the two opponent fish to acclimate and occupy the brick structure within its half of the arena without disturbance from its neighbor, during a period of three hours.

To start the contest trial, the experimenter removed the opaque divider from a position where she could not be seen by the fish. The mesh divider was still kept in place, but fish could start to interact visually. After one minute, a small dark board was attached to a corner of the arena as shelter for the subdominant fish, and the mesh divider was removed. Now, the previously subdivided brick construction constituted one single structure, requiring the fish to establish dominance and resolve the possession of this ‘territory’.

Videotaping of trials (camera model: Sony DC-SX30) started ca. two minutes before removal of the opaque board. Trials were also watched by the experimenter, who remained at a position unseen by the fish, in order to be able to stop the trial once a stable dominance hierarchy had been established or prematurely in case of enduring aggression. Since subordinate fish retreated to a corner of the arena and could hide in the provided shelter, whereas dominant fish stayed near the brick structure, no interruptions of the trials were necessary and no injuries occurred.

Most contests were resolved quickly, either after a short chase and a few charges against the subordinate fish, or after measuring each other’s strength by mouthfighting (opponents grip each other’s mouth and push) and circling. Trials were terminated when the subordinate fish (‘loser’) had retained the submissive colouration (reduced intensity, Fig. S1) and stopped challenging the dominant fish (‘winner’) for at least three minutes.

Video recordings were viewed in MPC-HC (Version 1.7.10) and time to resolution (competition duration) was measured as the interval between the first interaction after removal of the mesh divider and the start of the winner’s unchallenged dominance.

17 male-male contests and 18 female-female contests were staged between March and May 2016. Each fish participated in one contest. Two to four contests were carried out per day between 11:00 and 13:00 hours.

Contest opponents were size matched (mean difference ± sd 0.17 ± 0.16 cm) in order to minimize the known effect of body size on competitive success (Odreitz and Sefc 2015). In contrast, opponents were selected to maximize the difference in bar width between them. Mean bar width (± sd) of fish used in contests was 35 ± 3% of SL; mean difference (± sd) between opponents was 4 ± 3% of SL. Body sizes of the fish used in contests ranged from 7.2 – 10.4 cm SL, with mean SL (± sd) of 8.70 ± 0.78 cm.

**Melanophore density in black and yellow body regions**

A male *Tropheus* ‘Ikola’ was immersed in a solution of 500 mg/L epinephrine for 10 minutes to induce melanosome aggregation and sacrificed with an overdose of MS-222. Photographs were taken using a digital microscope (VHX-5000, Keyence Ltd.) with reflected light (figure S4).

**Data analysis**

All calculations were done in R vs. 3.1.2.

Size differences between opponents were calculated as RSD = (SL_focal fish_ – SL_opponent fish_) / (SL_focal fish_ + SL_opponent fish_). Similarly, bar width (BW) differences were calculated as RBD = (BW_focal fish_/SL_focal fish_ – BW_opponent fish_/SL_opponent fish_) / (BW_focal fish_/SL_focal fish_ + BW_opponent fish_/SL_opponent fish_). Condition factor (CF) was estimated as residuals from a log(weight) against log(SL) regression (Fechhelm et al. 1995). Differences in condition factor were calculated as CFD = CF_focal fish_ – CF_opponent fish_ (since CF was represented by residuals, scaling the difference by the sum of the two fish was not meaningful).

To assess whether winners differed from losers in RBD, CFD and RSD, we calculated these values for each trial according to the above formulas with the winner as 'focal fish’ and the loser as ‘opponent fish’. Although fish were approximately size matched, we were still interested in whether the small differences in body size affected contest outcome. Three linear models were fit, one for each of the three variables measured (i.e. RBD, RSD and CFD; all mean-centered and scaled). Contest type (sex of contestants) was included as a predictor and the remaining two of the three variables were included as covariates (as well as their interaction with contest type). Significant intercepts in these models would indicate that winners differed from losers for each respective variable (relevelling for each sex). A significant effect of contest type would indicate that RBD (or RSD; or CFD) between winners and losers differed between male and female contests.

A logistic regression (generalized linear model with binomial error distribution: contest outcome ~ RBD + RSD + CFD; package lme4; Bates et al. 2015) was used to estimate the effect of RBD on the outcome of female-female contests while controlling for CFD and RSD. Given our experimental design, the designations of fish within a trial as focus fish and opponent fish are arbitrary. Therefore, we generated permutations of the dataset, in which each of the two rival fish was randomly assigned as either the ‘focal’ or ‘opponent’ fish. Contest outcome was scored as binomial variable, with 1 indicating that the focal fish was the winner and 0 indicating that the opponent fish was the winner. The effects of RBD, RSD and CFD on contest outcome were estimated in the full model (contest outcome ~ RBD + RSD + CFD). ΔAIC values were calculated by dropping each predictor from the model independently (e.g. ΔAIC_RBD_ = AIC_reduced model excl. RBD_ - AIC_full model_). This procedure was repeated >1000 times, each time using a new random assignment of ‘focal’ and ‘opponent’ for each fish in the contests. In order to avoid overly imbalanced data (where the randomly assigned focal fish were nearly all winners or losers), only datasets in which 7-11 of the 18 female focal fish were winners were retained in the analyses (N=731 datasets). Logistic regression curves were drawn based on the full model estimates for the effects of RBD on the probability of winning, while accounting for CFD and RSD.

A general linear model investigated the effects of RBD, RSD and CFD on the duration of male and female contests. Absolute values of RBD, RSD and CFD were used to represent the extent of asymmetry between contestants in a trial. Contest duration was square-root-transformed and outliers (2 female and 3 male contests > 2 min) were excluded. The full model tested for interactions between contest type (sex of contestants) on the one hand and RBD, RSD and CFD on the other hand. Non-significant interactions (with RSD and CFD) were dropped from the final model.

Using measurements taken repeatedly from 44 individual fish over a time period of up to ~600 days, we compared the variance of bar width within individuals over time to variance among individuals. Based on ANOVA, the proportion of variance among individuals was estimated by the unbiased statistic ω² (Keren and Lewis 1979).

**References**

Bates D., Maechler M., Bolker B., Walker S. (2015). Fitting Linear Mixed-Effects Models Using lme4. *Journal of Statistical Software*, 67(1), 1-48.<[doi:10.18637/jss.v067.i01](https://doi.org/10.18637/jss.v067.i01)>.

Fechhelm R. G., Griffiths W. B., Wilson W. J., Gallaway B. J., Bryan J. D. (1995). Intra‐and interseasonal changes in the relative condition and proximate body composition of broad whitefish from the Prudhoe Bay region of Alaska. *Transactions of the American Fisheries Society*, *124*(4), 508-519.

Keren G., & Lewis C. (1979). Partial omega squared for ANOVA designs. *Educational and Psychological Measurement*, *39*(1), 119-128.

Odreitz U., Sefc K.M. (2015). Territorial competition and the evolutionary loss of sexual size dimorphism. *Behavioral Ecology and Sociobiology*, 69: 593-601.

**Supplementary Figures**

***Figure S1. Experimental arena.***

**
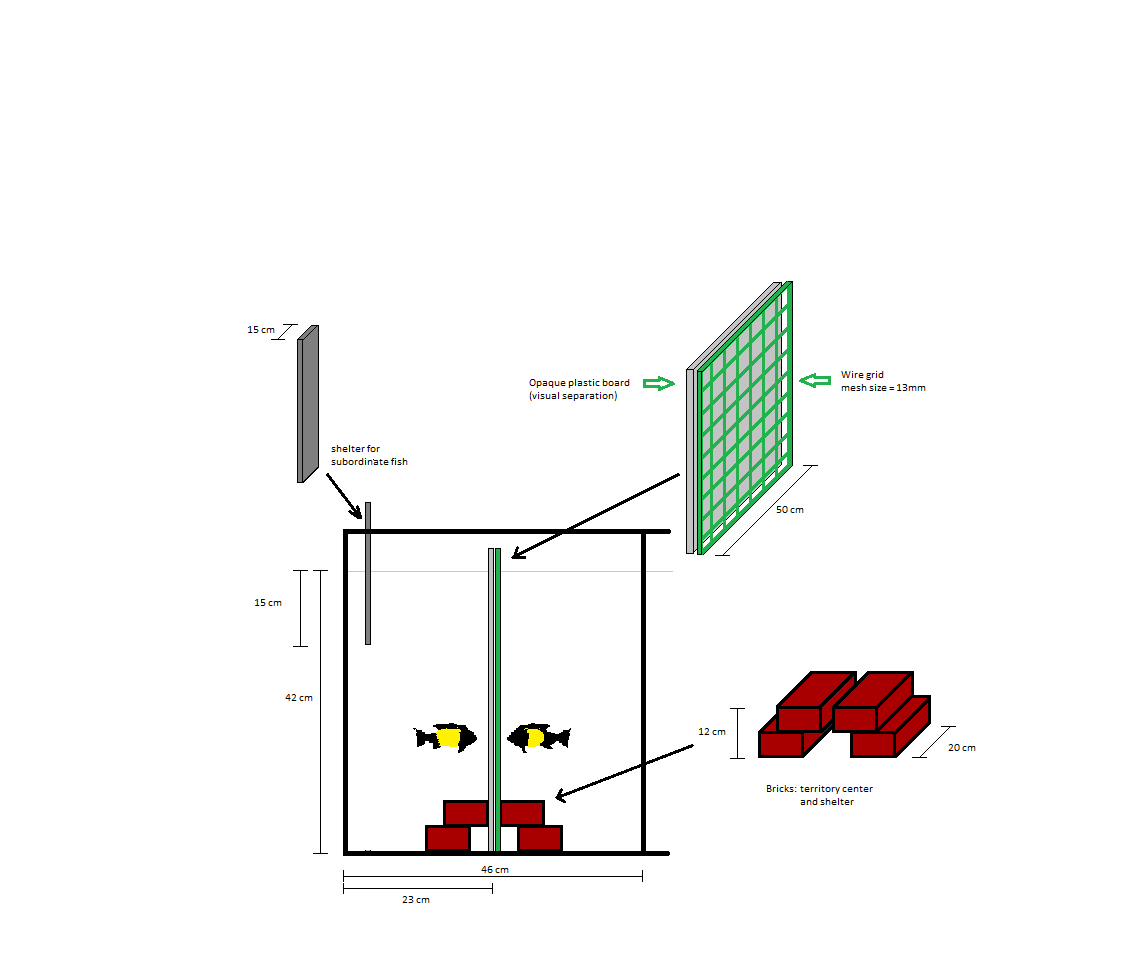
Fig. S2. Signaling by physiological colour change.**

The dominant fish (below) shows its maximal possible bar width in bright yellow colouration. Before contact with the lower fish, the upper fish had shown the same bright contrasts. Within seconds after contact, the upper fish reduced the intensity of both yellow and black colouration. Additionally, melanophores overlaying the xanthophores in the bar area were dispersed. When in extreme subordinate coloration, fish appear light grey from the head to the caudal peduncle.


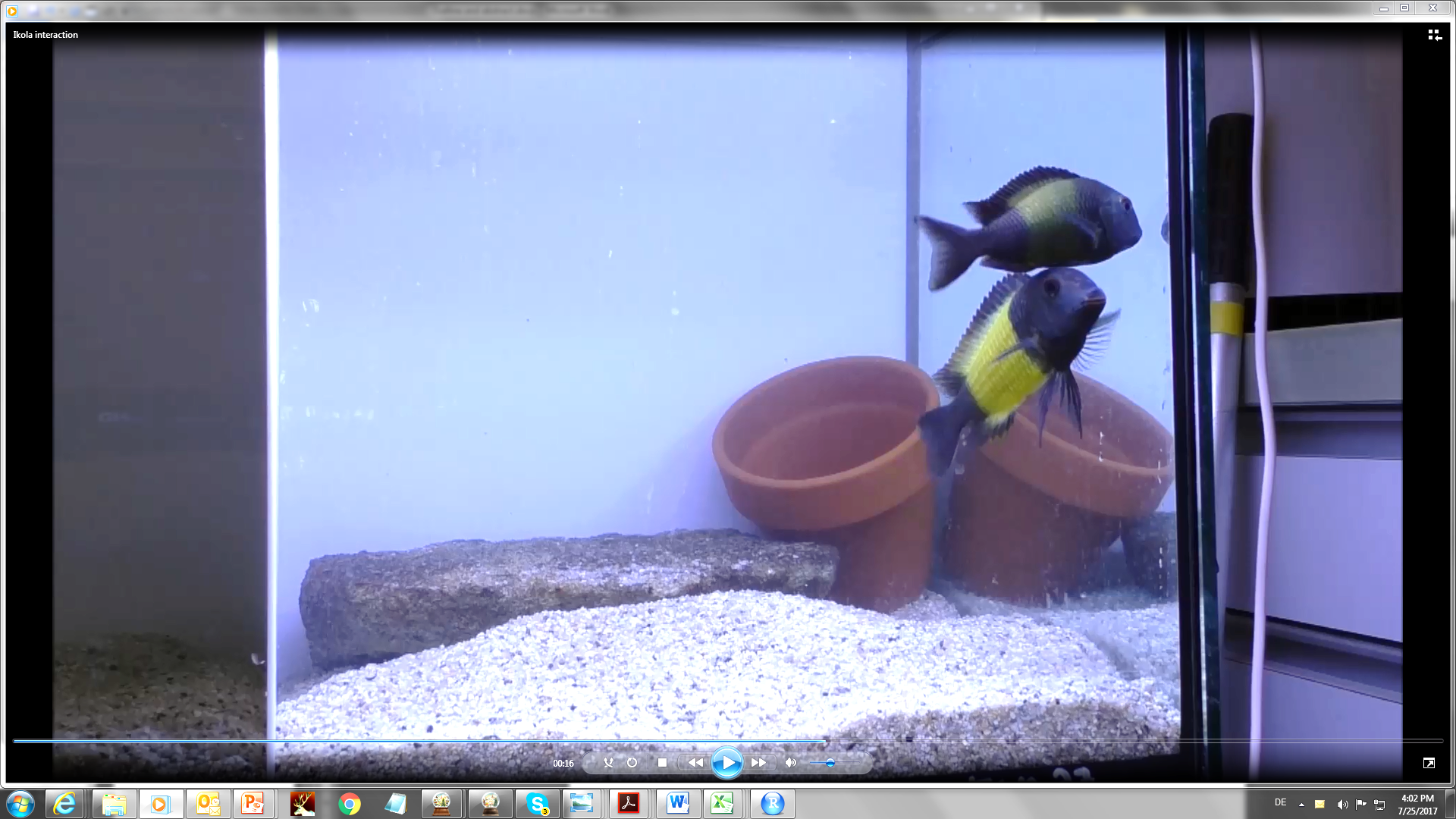


**Figure S3. Intra- and inter-individual variation in the width of the yellow bar.**

Photographs taken repeatedly from 44 individual fish over a time period of up to ~600 days were used to monitor intra-individual variation in relative bar width (bar width scaled by standard length, SL) across time. Bar width and SL were measured from photos showing the left side of the fish.

Below, each panel represents an individual fish (f and m numbers identify females and males, respectively), with bar width (blue dots) plotted against time. For each fish, its first photo represents time = 0. Dotted horizontal lines mark the upper and lower bounds of the variation displayed within each individual across time.


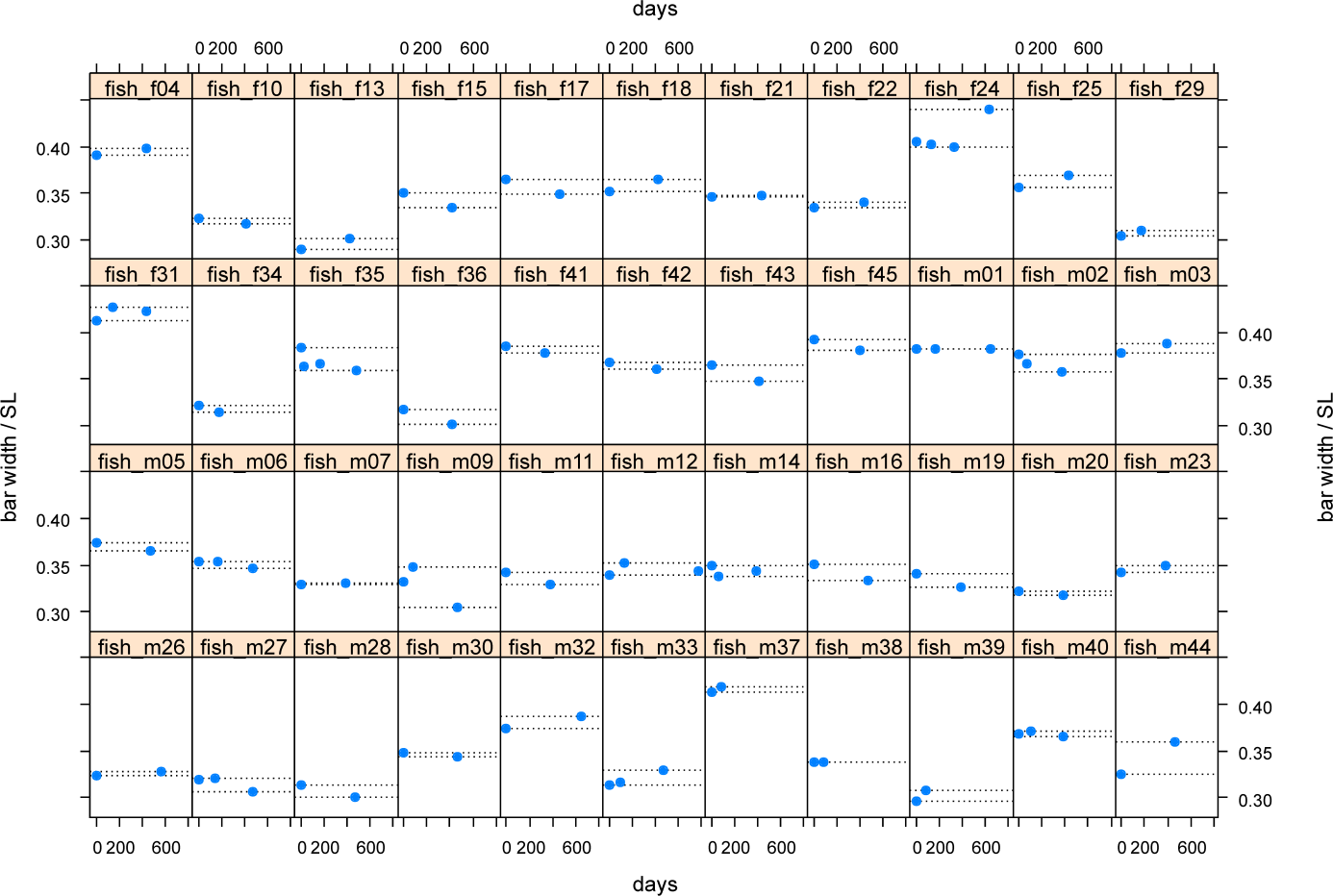


**Figure S4. Density of melanophores in black and yellow coloured body regions of *Tropheus* ‘Ikola’.**

The approximate positions of the body sections (A-D) are shown on the picture to the right (photo of a *Tropheus* ‘Ikola’; black line: width of the yellow bar).


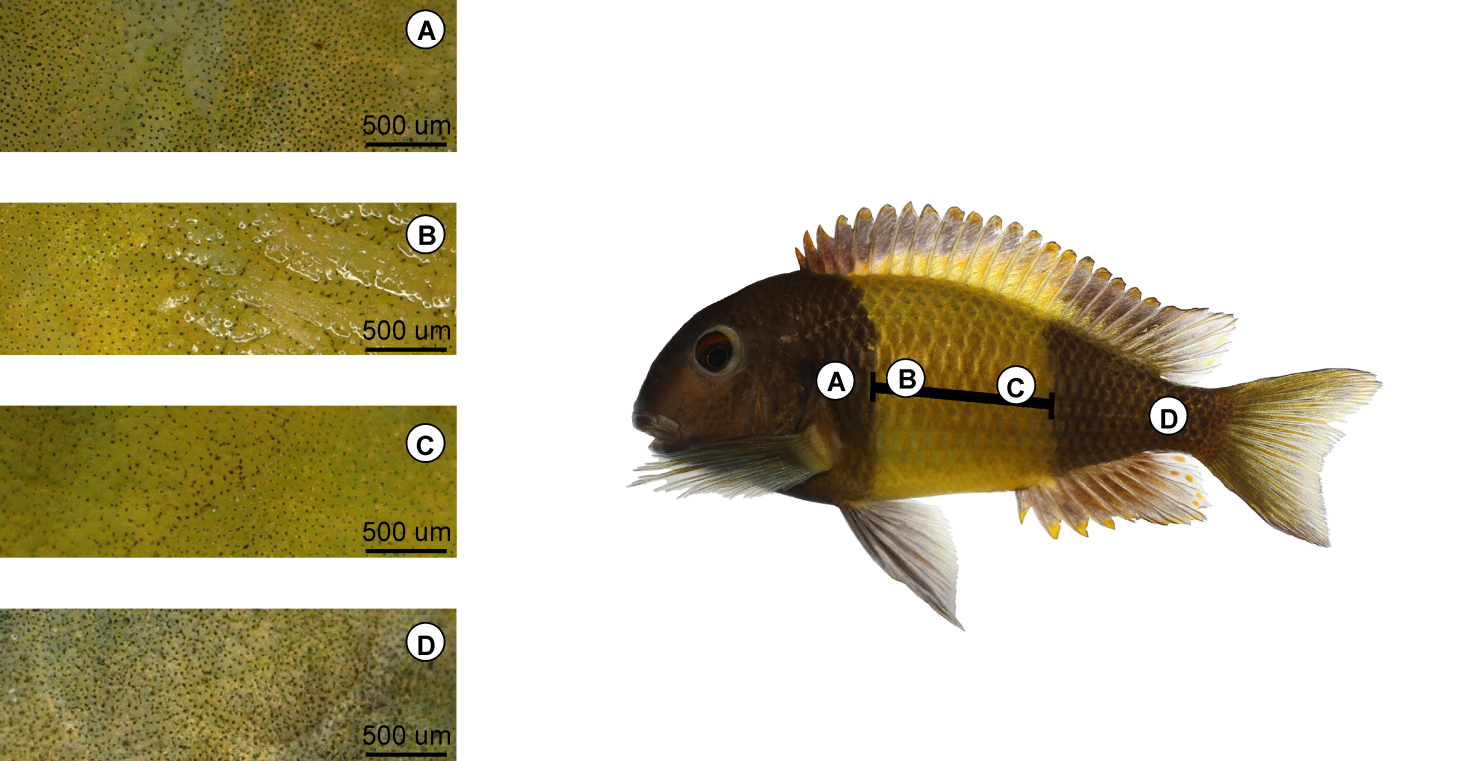

Supplement: Methods and supplementary figures [file rsbl20180480supp1.docx]
